# Irrelevant Task Difficulty Modulates the Emergence of Task Conflict

**DOI:** 10.5334/joc.475

**Published:** 2026-01-07

**Authors:** Ronen Hershman, Eldad Keha, Lisa Beckmann, Avishai Henik, Ayelet Sapir

**Affiliations:** 1 Department of Psychology, University of Innsbruck, Innsbruck, Austria; 2 Department of Psychology, The Hebrew University, Jerusalem, Israel; 3 Department of Psychology, Achva Academic College, Beer-Tuvia, Israel; 4 Department of Psychology, Ludwig-Maximilians-Universität München, Munich, Germany; 5 Department of Psychology and The Zelman Center for Brain Science, Ben-Gurion University of the Negev, Beer-Sheva, Israel; 6 School of Human Sciences, University of Greenwich, London, United Kingdom

**Keywords:** Automaticity, numerical cognition, Stroop effect, task conflict, cognitive control

## Abstract

In cognitive control tasks, participants are typically instructed to respond to a task-relevant dimension of a stimulus while ignoring the task-irrelevant one(s). In such experiments, task conflict reflects the additional effort associated with performing two tasks, such as identifying the color while reading the word in the color-word Stroop task. Task conflict is commonly inferred by comparing conditions that consist of two tasks (e.g., congruent and incongruent trials) with conditions that only consist of one task (meaningless non-word neutral trials). In three experiments, we used a color-digit Stroop task that varied in the difficulty of the irrelevant dimension of the stimuli, with these differences explicitly examined in a separate control experiment. While information conflict was evident across all experiments, we found differences in task conflict, so the harder it was to perceive the task-irrelevant dimension, the stronger the task conflict became. These findings demonstrate for the first time that task conflict emerges on a continuum, scaling with the level of engagement or processing demands associated with the irrelevant task. Moreover, these results suggest that our ability to inhibit the involuntary activation of an unwanted process is restricted. Therefore, despite the resource-intensive demands of completing the irrelevant task, it still takes place.

## 1. Introduction

### 1.1. The color-word Stroop task

The Stroop task, introduced by Stroop (1935), is a widely used cognitive control task that examines the ability to concentrate on relevant information while inhibiting irrelevant information. In this task, participants are presented with words in color and are instructed to quickly and accurately respond to the colors of the ink in which the words are written. There are three main conditions in the Stroop task: A congruent condition involves words whose meaning matches the color of the ink (e.g., the word “BLUE” presented in blue), an incongruent condition consists of words where the meaning conflicts with the presented color (e.g., the word “RED” presented in blue), and a neutral condition that includes stimuli such as non-color words (e.g., “TABLE”) or other meaningless stimuli like letter strings, pseudo-words, symbols, or colored patches. Interestingly, it was suggested that the meaning of neutral stimuli can influence task performance (Hershman et al., 2020, 2021; Kinoshita et al., 2017; Levin & Tzelgov, 2016). Typically, incongruent trials yield the longest reaction times (RTs), congruent trials result in the shortest RTs, and neutral trials fall somewhere in between. This leads to an interference effect, with slower responses on incongruent trials compared to neutral trials, as well as a small and fragile facilitation effect, where congruent trials are slightly faster than neutral trials (Henik et al., 2018; Parris et al., 2022).

Two types of conflicts are of interest in the Stroop task (Littman et al., 2019; Parris et al., 2023): information conflict and task conflict. *Information conflict* arises from contradictory information presented in the stimulus, such as the conflict between the meaning of the words and their ink color. This is typically explored by comparing incongruent trials (with conflicting information) to congruent trials (with matching information). *Task conflict* involves a conflict between relevant and irrelevant tasks associated with the stimulus. For instance, in the color-word Stroop task, the relevant task may involve responding to the color of the stimulus, while the irrelevant task may be reading the word. One of the early and influential demonstrations of task conflict came from Monsell et al. (2001), who showed that even non-color words or pronounceable non-words could interfere with color naming, suggesting that interference can arise from the mere activation of the word-reading task set, independently of specific response competition. Following studies measured task conflict by comparing congruent trials, where two possible tasks are triggered, to non-word neutral trials, where only one relevant task is triggered (Goldfarb & Henik, 2007; Hershman, Dadon, et al., 2024; Hershman & Henik, 2019; Kalanthroff & Henik, 2013). Under certain conditions, this may result in longer RTs for congruent compared to non-word neutral trials – the reverse facilitation effect (Kalanthroff et al., 2018; Keha & Kalanthroff, 2023). Conditions that reveal reverse facilitation often include highly sensitive measurements such as brain activation (Aarts et al., 2009; Bench et al., 1993; Carter et al., 1995) or changes in pupil size (Hershman, Dadon, et al., 2024; Hershman et al., 2020; Hershman & Henik, 2019, 2020). In RT experiments, reverse facilitation is observed when the expectation for conflict is low (Goldfarb & Henik, 2007; Keha & Kalanthroff, 2023), when control fails as in a stop-signal task (Kalanthroff et al., 2013), and when preparation time is reduced in a task-switching task (Kalanthroff & Henik, 2014), or in a highly constrained situations where confounding frequency effects are controlled (Spinelli & Lupker, 2025; Spinelli & Sulpizio, 2025); suggesting that the effect is seen under conditions of cognitive control relaxation. However, it should be noted that some previous findings have called into question the robustness and specificity of neural and behavioral indices associated with task conflict (Algom & Chajut, 2019; Parris et al., 2023). For example, Anterior Cingulate Cortex (ACC) activation, commonly linked to task conflict, may in some cases reflect broader contextual demands or trial-type uncertainty rather than conflict per se (Aarts et al., 2009; Floden et al., 2011; Parris et al., 2023).

### 1.2. The Gestalt color-word Stroop task

Recently, we have introduced the Gestalt version of the color-word Stroop task (Hershman, Sapir, et al., 2024). In the Gestalt color-word Stroop task, participants are presented with colored horizontal slices and are required to respond to their colors (as in the original Stroop task). In this task, the neutral trials include no other properties except the colored horizontal slices (Hershman et al., 2021, 2022), while in both the congruent and the incongruent trials, the colored horizontal slices include missing colored pixels, which, by applying the Gestalt principles (i.e., closure and figure/ground; Todorovic, 2008), could be perceived as words. It means that the task-irrelevant words are not presented explicitly, and a greater mental effort is required to engage with the task of reading. In line with previous color-word Stroop studies, an indication for the semantic processing of the Gestaltic words was found in the form of information conflict; that is, slower responses for incongruent trials than for congruent trials. Interestingly, in this task (Hershman, Sapir, et al., 2024), reverse facilitation was found, so that slower responses were observed for both congruent and incongruent trials than for neutral trials. It was suggested that, contrary to intuition, when the readability of the task-irrelevant stimuli is limited, participants do not ignore the task-irrelevant stimuli (Hershman, Sapir, et al., 2024). Instead, they exert more cognitive effort to process them, which results in reverse facilitation. This interpretation aligns with neuroimaging findings showing that visually degraded words engage more effortful processing, recruiting dorsal and fronto-parietal pathways, rather than the automatic ventral stream (Cohen et al., 2008). Thus, even when readability is reduced, the brain continues to process task-irrelevant stimuli, possibly with increased cognitive load, contributing to the reverse facilitation effect.

The idea that task difficulty, and particularly the difficulty of the reading task, enhances engagement with the task is not new. Research on the disfluency effect suggests that making reading more difficult (such as by using harder-to-read fonts), can lead to deeper cognitive processing and better learning outcomes under certain conditions (Seufert et al., 2017). According to disfluency theory, difficult-to-read materials serve as metacognitive cues that increase cognitive engagement, prompting individuals to shift from an intuitive, fast-processing mode to a more deliberate, analytic mode (Alter et al., 2007). Empirical studies have demonstrated that moderate levels of disfluency improve recall, comprehension, and transfer by fostering deeper processing, whereas extreme levels of disfluency can impair performance by overloading extraneous cognitive load (Diemand-Yauman et al., 2011; Seufert et al., 2017). Notably, the relationship between disfluency and performance follows a reversed U-shaped curve, in which learning benefits are maximized at intermediate levels of difficulty but decline when the task becomes excessively hard (Oppenheimer & Alter, 2014). The findings regarding the moderate levels of disfluency align with the findings of the Gestalt color-word Stroop task, in which the task-irrelevant words are less explicitly visible due to missing pixels. Rather than being ignored, this perceptual challenge paradoxically enhances engagement with the task-irrelevant dimension. It was proposed that the ease or difficulty of reading determines the type and extent of conflict that arises during a task (Hershman, Sapir, et al., 2024). When reading is easy, it occurs automatically, leading primarily to information conflict with relatively low task conflict, as processing the irrelevant reading task does not require substantial effort. This is the case in the classic color-word Stroop task, where clearly visible words are effortlessly processed, and the conflict arises mainly from the mismatch between the meaning of the words and the task goal of color naming. However, when reading is challenging but still possible, as in the Gestalt Stroop task, the harder it is to process the words, the greater the task conflict. This occurs because attempting to resolve the ambiguity of the degraded words requires additional cognitive resources, increasing the overall demand of the task and heightening both task conflict and information conflict. Supporting this idea, prior research suggested that individuals who require greater cognitive effort to process an irrelevant reading task—such as participants with dyslexia (Protopapas et al., 2007) or participants reading in a second language (Datta et al., 2019; Tzelgov et al., 1990)—experience greater interference effects in the Stroop task.

The aim of the current study was to further examine whether different levels of difficulty in the processing of the irrelevant dimension will result in different levels of task conflict. Unlike prior research, which has focused on how disfluency affects performance in the relevant dimension (e.g., reading comprehension), our findings suggest that similar effects can occur in the irrelevant dimension. When the readability of task-irrelevant stimuli is compromised, cognitive resources are recruited to process their meaning despite their irrelevance to the primary task. This suggests a novel extension of the disfluency effect: not only can task difficulty enhance engagement with relevant stimuli, but perceptual constraints can also amplify attention to task-irrelevant information, thereby yielding a reverse facilitation effect, which serves as a marker for task conflict. In the current experiments, we used the color-digit Stroop task (Hershman, et al., 2025b) while varying the difficulty of the task-irrelevant dimension using Gestalt principles.

### 1.3. The color-digit Stroop task

In the color-digit Stroop task (Hershman, Keha, et al., 2024; Hershman et al., 2025a, 2025b), participants are presented with colored sliced stimuli and are required to respond to the number of the presented colors. Similar to other Stroop-like tasks, the color-digit Stroop task includes three congruency conditions: congruent in which the colored slices create a digit that represents the number of presented colors (e.g., the digit ‘3’ that consists of slices with three colors), incongruent in which the colored slices created a digit representing a number different than the number of the presented colors (e.g., the digit ‘3’ consisting of slices with four colors), and neutral in which the colored slices did not create any meaningful object or symbol (Hershman et al., 2021, 2022).

That is, incongruent trials were slower than neutral trials, which were slower than congruent trials. In addition, in line with previous Stroop and pupillometry studies (Hershman, Dadon, et al., 2024; Hershman et al., 2020; Hershman & Henik, 2019, 2020), when changes in pupil sizes were examined in the color-digit Stroop task, both congruency effect (less pupil dilation for congruent trials than for incongruent trials) and reverse facilitation (Hershman et al., 2025a) were revealed. This pattern provides evidence for both information and task conflicts in the color-digit Stroop task.

### 1.4. The present study

Across three experiments, we varied the difficulty of perceiving the numerical value of the presented digits (the differences in difficulty were examined and explicitly verified in a separate control experiment – Exp. 4). These digits were formed by using Gestalt principles. In the fourth experiment, we explicitly examined the perceptual difficulty levels of the stimuli. In the first three experiments, participants were presented with colored slices and were asked to report the number of colors in the stimuli. In Exp. 1, these color slices were superimposed with a single digit at the same color as the white background (see Table 1), which was relatively easy to perceive. In Exp. 2, we used Hebrew number words and sliced them into colors (see Table 3). In Exp. 3, similar to Hershman et al.’s Gestalt Stroop task (Hershman, Sapir, et al., 2024), the color slices were cut in a way that the Hebrew number words could be perceived only when using Gestalt principles. Importantly, the neutral trials in all the experiments were the same; color slices without secondary numerical meaning (Hershman et al., 2021, 2022). We expected that as the processing difficulty of the task-irrelevant numerical value increases, the evidence for task conflict would be observed.

**Table 1:** Examples of Stimuli that Were Used in Exp. 1.


CONDITION	1 COLOR	2 COLORS	3 COLORS	4 COLORS

Congruent	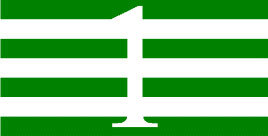	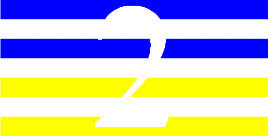	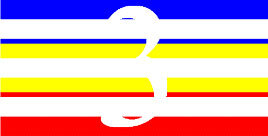	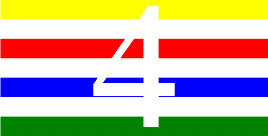

Neutral	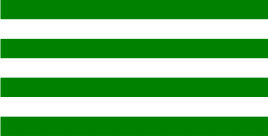	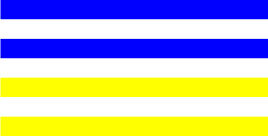	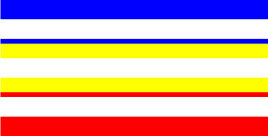	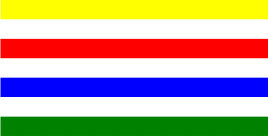

Incongruent	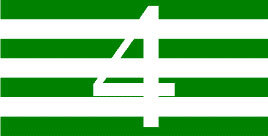	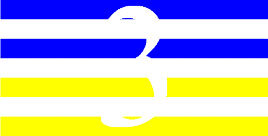	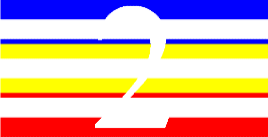	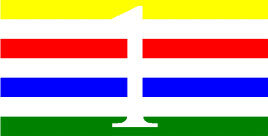


*Note*. For the neutral condition, horizontal colored slices containing 1–4 colors were used. For both the congruent and incongruent conditions, an additional white single digit (1–4) was embedded at the center of the colored slices (the entire set of stimuli can be downloaded from https://osf.io/f27j8/?view_only=c94d9d6d718d4883b30f991c8a2cd1f0).

## 2. Experiment 1

### 2.1. Method

#### 2.1.1. Participants

Based on the effects in Hershman, Beckmann, et al., (2025b), we computed the required sample size for an expected effect size of partial eta squared = .157 (corresponding to a Cohen’s f = 0.431), for 3 experiments × 2 (congruency: congruent and neutral) mixed-model ANOVA design. With α set at .05 and desired power at .95, the analysis indicated that a total of 90 participants (30 per experiment) would be required to detect the predicted interaction. Accordingly, we set our target sample size to include at least 30 valid participants per experiment. However, since the experiment was conducted online, we anticipated a larger number of submissions, and therefore, we recruited a larger number of participants.

Forty-three participants (37 females & 6 males, mean age 24.67 years, *SD* = 4.67) from Achva Academic College participated in the experiment in return for course credit. The study was approved by the ethics committee of the Psychology Department of Ben Gurion University. All participants had no reported history of attention deficit disorder, learning disabilities, or color blindness.

#### 2.1.2. Stimuli

Participants were presented with colored rectangles in a size of 357 × 551 pixels, cut into four horizontal slices. In the center of the colored rectangles, a white single-digit numbers (1, 2, 3, and 4) in Times New Roman font were concealed. These stimuli were created following the same approach as in Hershman et al.’s stimuli (Hershman, Sapir, et al., 2024), but here, the stimuli were numerical digits rather than words. The colored rectangles could consist of 1–4 colors (red, blue, green, and yellow; see examples of the stimuli in Table 1). Three possible congruency conditions were created; the number of colors could have been congruent with the numerical value of the single-digit number (e.g., the number 3 against three colors) or incongruent (e.g., the number 3 against two colors). The neutral conditions consisted of the same colored slices without a number. In total, there were 64 neutral stimuli, 64 congruent stimuli, and 192 incongruent stimuli across all combinations of digits and color conditions. The conditions and the stimuli for each participant were selected randomly from a pool that included all the possible combinations. The presented stimuli appeared against a white (RGB: 255, 255, 255) background.

#### 2.1.3. Procedure

**Figure 1:**
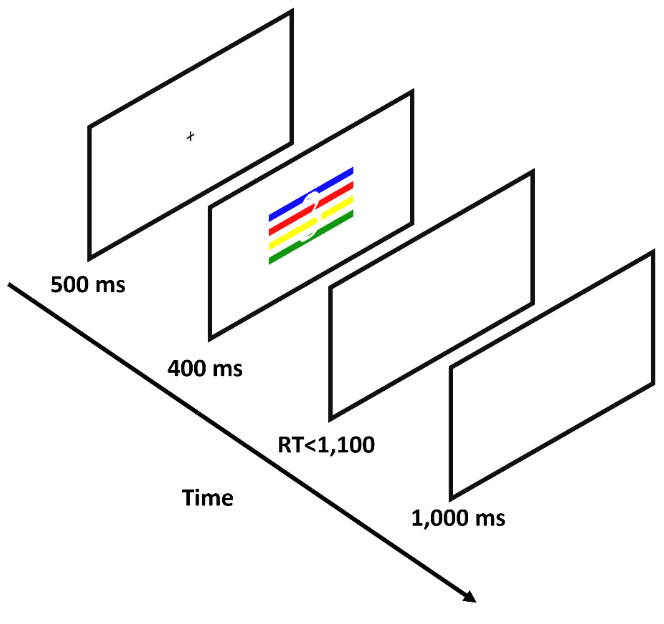
Schematic Representation of an Experimental Trial. *Note*. Participants were asked to indicate how many colors were present on the displayed stimulus (four in the example shown).

Participants were tested online by using minnoJS (Zlotnick et al., 2015) on their own devices. The program required a spacebar response, ensuring participants only use computers rather than tablets or mobile phones. The experiment included 12 practice trials that were excluded from the analysis. After each practice trial, participants received feedback on their accuracy. Participants had to achieve at least 80% correct trials to proceed to the experimental part (i.e., at least ten correct responses); otherwise, they had to repeat the practice. In the experimental part, participants carried out 432 trials; 144 for each congruency condition. A trial began with 500 ms of a black (RGB: 0, 0, 0) fixation cross at the center of the screen. The fixation was followed by a visual stimulus that appeared on the screen for 400 ms, and was followed by a blank screen for a maximum of 1,100 ms, or until a key-press. Each trial ended with a 1,000 ms inter-trial interval (ITI) of a blank screen (see Figure 1). Participants were asked to respond using both hands and press the “Z” key if the stimulus contained one color, the “X” key if the stimulus contained two colors, the “N” key if the stimulus contained three colors, and the “M” key if the stimulus contained four colors. This is a standard horizontal arrangement of responses that is used in Stroop and Stroop-like tasks (Hershman, et al., 2025b; Hershman et al., 2020; Hershman & Henik, 2019). RT was calculated from the appearance of the visual stimulus to the reaction in the form of a key-press.

### 2.2. Results

One participant was excluded from the analysis due to an accuracy of less than 50% in each condition. For each of the remaining 42 participants (35 females & 7 males, mean age = 24.67 years, SD = 4.71). The data were analyzed using JASP (JASP Team, 2025).

#### 2.2.1. Error rates

The mean error rates for each participant in each condition were subjected to a one-way repeated-measures analysis of variance (ANOVA) with congruency (congruent, incongruent, and neutral) as an independent factor (mean error rates in the various conditions are presented in Table 2). The analysis produced a meaningful (*BF*_10_ ≥ 3) main effect for congruency, *F*(2,82) = 21.94, *p* <. 001, \[
\eta _{p}^{2}\] = .35, *BF*_10_ > 10^5^. Mean error rates in incongruent trials were higher than in neutral trials, *t*(41) = 4.65, *p_holm_* < .001, *BF*_10_ > 100, *Cohen’s d* = 0.72, and then in congruent trials, *t*(41) = 6.40, *p_holm_* < .001, *BF*_10_ > 10^5^, *Cohen’s d* = 0.99. No differences were found between congruent and neutral trials *t*(41) = 1.46, *p_holm_* = .123, *BF*_10_ = 0.45 ≡ *BF*_01_ = 2.23, *Cohen’s d* = 0.23.

**Table 2:** Mean Reaction Time and Error Rates for Each Congruency Condition in Experiments. 1–4.


STIMULUS TYPE	MEAN RT (MS)	95% CI (MS)	ERROR RATES (%)	95% CI (%)

Exp. 1				

Congruent	585	556–614	3.76	2.43–5.09

Neutral	596	568–624	4.33	2.73–5.94

Incongruent	637	602–672	6.33	4.95–7.72

Exp. 2				

Congruent	605	580–630	12.35	9.01–15.69

Neutral	602	577–627	11.07	8.11–14.04

Incongruent	640	609–672	17.30	13.93–20.68

Exp. 3				

Congruent	637	610–663	14.14	10.94–17.33

Neutral	618	594–643	12.62	9.54–15.70

Incongruent	655	626–683	17.56	14.39–20.80

Exp. 4				

Digits	560	526–594	18.93	11.08–26.78

Words	602	567–637	21.83	13.96–29.69

Gestalt words	637	601–673	27.90	19.34–35.86


*Note*. RT = reaction time. CI represents 95% confidence intervals.

#### 2.2.2. Reaction Times

Mean RT and standard deviation were calculated across all the experimental trials. Then, trials that were 2.5 z-scores faster or slower than the mean were excluded from the analysis. Mean RTs of correct response trials for each participant in each condition were subjected to a one-way repeated-measures ANOVA with congruency (congruent, incongruent, and neutral) as an independent factor (mean RTs in the various conditions are presented in Table 2). The analysis produced a meaningful (*BF*_10_ ≥ 3) main effect for congruency, *F*(2,82) = 80.93, *p* < .001, \[
\eta _{p}^{2}\] = .664, *BF*_10_ > 10^16^. Mean RT in incongruent trials was longer than in neutral trials, *t*(41) = 7.87, *p_holm_* < .001, *BF*_10_ > 10^7^, *Cohen’s d* = 1.21, which was longer than congruent trials *t*(41) = 4.63, *p_holm_* < .001, *BF*_10_ = 604, *Cohen’s d* = 0.72. Transitively, the mean RT in incongruent trials was longer than in congruent trials, *t*(41) = 10.91, *p_holm_* < .001, *BF*_10_ > 10^10^, *Cohen’s d* = 1.68.

A post hoc power analysis was conducted for the main effect of congruency in Experiment 1, based on a repeated-measures ANOVA (within-subjects) with three levels of congruency, α = .05, total sample size = 42, and an observed effect size of Cohen’s *f* = 1.41 (\[
\eta _{p}^{2}\] = .664). The achieved power (1–β) was 99.99%, indicating that the study was fully powered to detect the observed effect in Experiment 1.

### 2.3. Discussion

In this experiment, we ran an alternative version of the color-digit Stroop task (Hershman, et al., 2025a, 2025b). Participants were presented with colored slices and were asked to determine, as quickly as possible, how many colors were presented. In the center of the colored slices, in the same color as the background, a single digit might have been perceived. In trials in which the numerical value of the digit was congruent with the number of presented colors (e.g., the number 3 against three colors), responses were faster than in neutral trials when no digit was presented. These neutral trials were faster than trials in which the numerical value of the perceived digit was incongruent with the number of presented colors (e.g., the number 3 against two colors). In other words, both facilitation and interference were observed in this task. These results replicate the results that were previously observed in the color-digit Stroop task (Hershman, et al., 2025a, 2025b).

In contrast to the results in the Gestalt Stroop task (Hershman, Sapir, et al., 2024), no reverse facilitation was observed (although the same Gestalt principles of closure and figure/ground (Todorovic, 2008) were used). Therefore, it could be argued that the processing of the irrelevant dimension (i.e., the numerical value of the single digits) was not difficult to perceive compared to the neutral condition, which did not require the processing of irrelevant pieces of information. Accordingly, we posit that an increment in the difficulty level of the processing is required to reveal the reverse facilitation and, thus, task conflict. For this reason, we designed another version of the color-digit Stroop task in which the processing of the irrelevant dimension was expected to be less accessible. If the difficulty of the irrelevant task impacts the reverse facilitation, the prediction would be that RTs to congruent trials will not be shorter compared to neutral trials.

## 3. Experiment 2

In this experiment, we replaced the single-digit numbers with Hebrew number words. That is, words that indicate a numerical value. Similar to Hershman et al.’s original color-digit Stroop study (Hershman, et al., 2025a), we used horizontal slices that were removed from the stimuli with the aim of making the words harder to read (see Table 3). Using stimuli that are harder to read, we anticipated that participants would exert more mental effort on the irrelevant reading task. This added difficulty should manifest as longer RTs on incongruent trials compared to neutral trials. Additionally, we expected a reduced difference between congruent and neutral trials or even a reversal pattern where neutral trials might show shorter RTs than congruent ones (a reverse facilitation effect).

**Table 3:** Examples of Stimuli that Were Used in Exp. 2.


CONDITION	1 COLOR	2 COLORS	3 COLORS	4 COLORS

Congruent	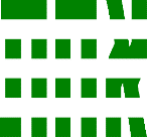	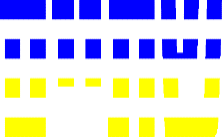	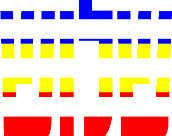	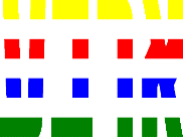

Neutral	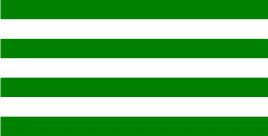	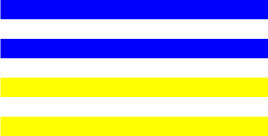	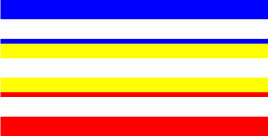	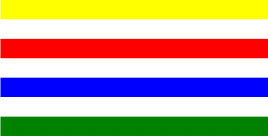

Incongruent	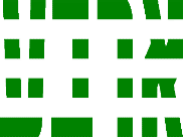	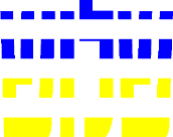	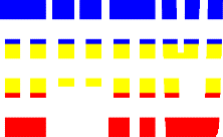	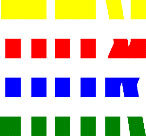

Incongruent (English example)	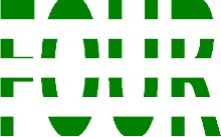	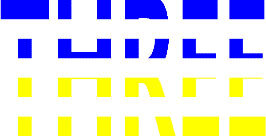	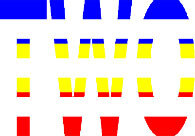	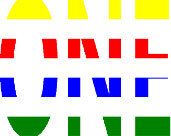


*Note*. For the neutral condition, horizontal colored slices containing 1–4 colors were used. For both the congruent and incongruent conditions, the Hebrew number words אחת (Akhat, “one”), שתיים (Shtaim, “two”), שלוש (Shalosh, “three”), and ארבע (Arba, “four”) were cut into horizontal slices that could each contain 1–4 colors (the entire set of stimuli can be downloaded from https://osf.io/f27j8/?view_only=c94d9d6d718d4883b30f991c8a2cd1f0).

### 3.1. Method

#### 3.1.1. Participants

Forty participants (37 females & 3 males, mean age 23.67 years, *SD* = 2.55) from Achva Academic College and Colman College of Management participated in the experiment in return for course credit. The study was approved by the ethics committee of the Psychology Department of Ben Gurion University. All participants had no reported history of attention deficit disorder, learning disabilities, or color blindness.

#### 3.1.2. Stimuli

Participants were presented with colored Hebrew number words (Aduma font). The words were אחת (Akhat – one), שתיים (Shtaim – two), שלוש (Shalosh – three), and ארבע (Arba – four) in a size of 357 × 551 pixels. The words were cut into slices that could contain 1–4 colors (red, blue, green, and yellow; see examples of the stimuli in Table 3).

Similar to Exp. 1 and in line with previous color-digit Stroop tasks (Hershman, et al., 2025a, 2025b), three congruency conditions were used: The number of colors could have been congruent with the numerical value of the presented word (e.g., the word THREE painted with three colors) or incongruent (e.g., the word TREE painted with two colors). In addition to the colored number words, participants were also presented with colored rectangles of the same size and colors as in Exp. 1, which were used as neutral stimuli. The conditions and the stimuli for each participant were selected randomly from a pool that included all the possible combinations. The stimuli appeared against a white (RGB: 255, 255, 255) background.

#### 3.1.3. Procedure

The procedure was identical to the one in Exp. 1.

### 3.2. Results

Five participants were excluded from the analysis due to low accuracy rates (less than 50% in each condition). Similar to Exp. 1, for each of the remaining 35 participants (32 females & 3 males, mean age = 22.54 years, SD = 8.36), mean RT and standard deviation were calculated across all the experimental trials. Then, outliers RTs that were 2.5 z-scores lower or higher than the mean of each subject were excluded from the analysis. The data were analyzed using JASP (JASP Team, 2025).

#### 3.2.1. Error Rates

The mean error rates for each participant in each condition were subjected to a one-way repeated-measures ANOVA with congruency (congruent, incongruent, and neutral) as an independent factor (mean error rates in the various conditions are presented in Table 2). The analysis produced a meaningful (*BF*_10_ ≥ 3) main effect for congruency, *F*(2,68) = 34.51, *p* < .001, \[
\eta _{p}^{2}\] = .5, *BF*_10_ > 10^8^. Mean error rates in incongruent trials were higher than in neutral trials, *t*(34) = 7.67, *p_holm_* < .001, *BF*_10_ > 10^6^, *Cohen’s d* = 1.3, and then in congruent trials, *t*(34) = 5.77, *p_holm_* < .001, *BF*_10_ > 10^4^, *Cohen’s d* = 0.98. No differences were found between congruent and neutral trials *t*(34) = 1.83, *p_holm_* = .08, *BF*_10_ = 0.8 ≡ *BF*_01_ = 1.24, *Cohen’s d* = 0.31.

#### 3.2.2. Reaction Times

Mean RTs of correct response trials for each participant in each condition were subjected to a one-way repeated-measures ANOVA with congruency (congruent, incongruent, and neutral) as an independent factor (mean RTs in the various conditions are presented in Table 2). A meaningful (*BF*_10_ ≥ 3) main effect for congruency was found, *F*(2,68) = 44.66, *p_holm_* < .001, \[
\eta _{p}^{2}\] =.57, *BF*_10_ > 10^10^. Specifically, mean RT in incongruent trials was longer than in neutral trials, *t*(34) = 7.63, *p_holm_* < .001, *BF*_10_ > 10^6^, *Cohen’s d* = 1.29, as well as in congruent trials, *t*(34) = 6.67, *p_holm_* < .001, *BF*_10_ > 10^5^, *Cohen’s d* = 1.13. However, no differences were found between congruent and neutral trials, *t*(34) = 1.18, *p* = .25, *BF*_10_ = 0.34 ≡ *BF*_01_ = 2.9, *Cohen’s d* = 0.2.

A post hoc power analysis was conducted for the main effect of congruency in Experiment 2, based on a repeated-measures ANOVA (within-subjects) with three levels of congruency, α = .05, total sample size = 35, and an observed effect size of Cohen’s f = 1.16 (\[
\eta _{p}^{2}\] = .57). The achieved power (1–β) was 99.99%, indicating that the study was fully powered to detect the observed effect in Experiment 2.

### 3.3. Discussion

In this experiment, we replaced the single-digit stimuli we used in Exp. 1 with Hebrew number words. Participants were presented with Hebrew number words, which indicated the numerical values of 1–4 and consisted of 1–4 colors. Participants were asked to determine the number of colors in the stimulus as quickly as possible. In line with Exp. 1 as well as previous color-digit Stroop studies (Hershman, et al., 2025a, 2025b), trials in which the numerical value of the Hebrew number word was incongruent with the number of colors (e.g., the word THREE which consisted of two colors), produced responses that were slower than both, trials in which the numerical value of the Hebrew number word was congruent with the number of colors (e.g., the word THREE which consisted of three colors), and neutral trials in which no word was presented. However, in contrast to Exp. 1, no facilitation was found; the RTs for neutral trials were not longer than those for congruent trials. This absence of facilitation effect in Exp. 2 might be explained by the fragility of the facilitation effect (Kalanthroff et al., 2013; MacLeod, 1991). Additionally—and more importantly—words are visually more complex than digits, which may have made them harder to process. As a result, participants likely expended more mental effort on the irrelevant reading task when exposed to non-neutral stimuli. This suggests that a more pronounced experimental condition, such as increased reading difficulty, could provide clearer evidence of task conflict, potentially revealed by a reverse facilitation effect.

## 4. Experiment 3

In Exp. 3, we tried to make the irrelevant dimension even harder to process. We used the same concept as Hershman et al.’s study task (Hershman, Sapir, et al., 2024), which used the Gestalt principles of closure and figure/ground (Todorovic, 2008). Specifically, instead of the explicit presentation of the Hebrew number words, we used an implicit presentation of them. As suggested (Hershman, Sapir, et al., 2024), this kind of stimulus makes the meaning of the irrelevant dimension more difficult to process. The prediction was, therefore, that trials in which the processing of the meaning of the irrelevant dimension would be possible (i.e., both congruent and incongruent trials) would require significantly more mental effort compared to neutral trials. In turn, this will result in reverse facilitation – longer RTs for congruent trials compared to neutral trials. In other words, we expected to find an indication of the presence of task conflict.

### 4.1. Method

#### 4.1.1. Participants

Thirty-eight participants (32 females & 6 males, mean age 22.92 years, *SD* = 1.48) from Ben-Gurion University of the Negev participated in the experiment in return for 20 NIS (about 5 USD). The study was approved by the ethics committee of the Psychology Department. None of the participants reported a history of attention deficit disorder, learning disabilities, or color blindness.

#### 4.1.2. Stimuli

Participants were presented with colored rectangles in a size of 357 × 551 pixels. Inside the colored rectangles, colored Hebrew number words (Aduma font) in the same color as the background were concealed, which could be perceived using the Gestalt principles (i.e., closure and figure/ground; Todorovic, 2008). The words that were used were אחת (Akhat – one), שתיים (Shtaim – two), שלוש (Shalosh – three), and ארבע (Arba – four). To make the perception of the stimuli less explicit, the stimuli were cut horizontally into four parts so that the gaps between these segments blended into the Hebrew word numbers. The colored slices could consist of 1–4 colors (red, blue, green, and yellow; see examples of the stimuli in Table 4). The number of colors could have been congruent with the numerical value of the word (e.g., the word THREE against three colors) or incongruent (e.g., the word THREE against two colors). As neutral trials, participants were presented with colored rectangles of the same size and colors as in Exps. 1 and 2, which carried no additional meaning. The conditions and the stimuli for each participant were selected randomly from a pool that included all the possible combinations. The presented stimuli appeared against a white (RGB: 255, 255, 255) background.

**Table 4:** Examples of Stimuli that Were Used in Exp. 3.


CONDITION	1 COLOR	2 COLORS	3 COLORS	4 COLORS

Congruent	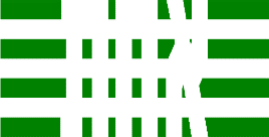	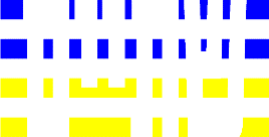	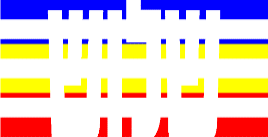	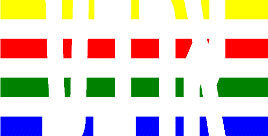

Neutral	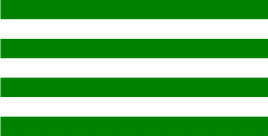	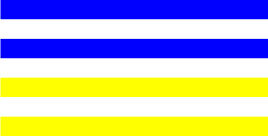	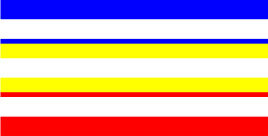	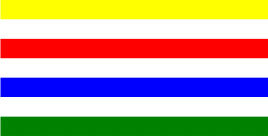

Incongruent	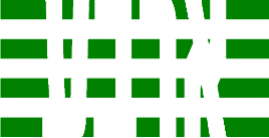	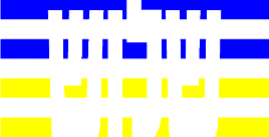	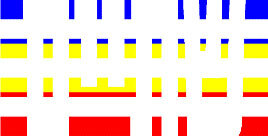	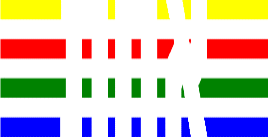

Incongruent (English example)	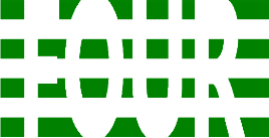	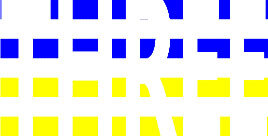	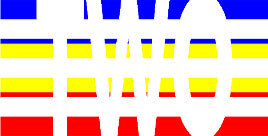	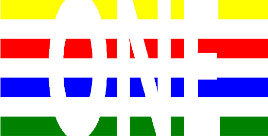


*Note*. For the neutral condition, horizontal colored slices containing 1–4 colors were used. For both the congruent and incongruent conditions, a white Hebrew number word (Akhat, “one”), שתיים (Shtaim, “two”), שלוש (Shalosh, “three”), and ארבע (Arba, “four”) was embedded at the center of the colored slices (the entire set of stimuli can be downloaded from https://osf.io/f27j8/?view_only=c94d9d6d718d4883b30f991c8a2cd1f0).

#### 4.1.3. Procedure

The procedure was identical to that of Exp. 1 and Exp. 2.

### 4.2. Results

Three participants were excluded from the analysis due to an accuracy lower than 50% in each condition. Similar to Exp. 1 & Exp. 2, for each participant (in total 35 participants; 29 females & 6 males, mean age = 23.03 years, SD = 1.46), mean RT and standard deviation were calculated across all the experimental trials. Then, RTs that were larger or smaller than 2.5 z-scores from the mean of each subject were excluded from the analysis. The data were analyzed using JASP (JASP Team, 2025).

#### 4.2.1. Error Rates

The mean error rates for each participant in each condition were subjected to a one-way repeated-measures ANOVA with congruency (congruent, incongruent, and neutral) as an independent factor (mean error rates in the various conditions are presented in Table 2). The analysis produced a meaningful (*BF*_10_ ≥ 3) main effect for congruency, *F*(2,68) = 13.87, *p* < .001, \[
\eta _{p}^{2}\] = .28, *BF*_10_ > 1,000. Mean error rates in incongruent trials were higher than in neutral trials, *t*(34) = 4.71, *p_holm_* < .001, *BF*_10_ > 100, *Cohen’s d* = 0.77, and then in congruent trials, *t*(34) = 3.37, *p_holm_* = .004, *BF*_10_ = 18.55, *Cohen’s d* = 0.55. No differences were found between congruent and neutral trials *t*(34) = 1.89, *p_holm_* = .08, *BF*_10_ = 0.88 ≡ *BF*_01_ = 1.14, *Cohen’s d* = 0.31.

#### 4.2.2. Reaction Times

Mean RTs of correct response trials for each participant in each condition were subjected to a one-way repeated-measures ANOVA with congruency (congruent, incongruent, and neutral) as an independent factor (mean RTs in the various conditions are presented in Table 2). As expected, our analysis produced a meaningful (*BF*_10_ ≥ 3) main effect for congruency, *F*(2,68) = 35.84, *p* < .001, \[
\eta _{p}^{2}\] = .51, *BF*_10_ > 10^8^. Specifically, mean RT in incongruent trials was longer than in neutral trials, *t*(34) = 7.14, *p_holm_* < .001, *BF*_10_ > 10^5^, *Cohen’ s d* = 1.20, and in congruent trials, *t*(34) = 4.23, *p_holm_* < .001, *BF*_10_ = 158.62, *Cohen’ s d* = 0.72. In addition, congruent trials were longer than neutral trials, *t*(34) = 5.49, *p_holm_* < .001, *BF*_10_ > 1,000, *Cohen’ s d* = 0.93, indicating a reversed facilitation.

A post hoc power analysis was conducted for the main effect of congruency in Experiment 3, based on a repeated-measures ANOVA (within-subjects) with three levels of congruency, α = .05, total sample size = 35, and an observed effect size of Cohen’s f = 1.02 (\[
\eta _{p}^{2}\] = .51). The achieved power (1–β) was 99.99%, indicating that the study was fully powered to detect the observed effect in Experiment 3.

### 4.3. Discussion

In this experiment, we used the procedure of Hershman et al.’s study (Hershman, Sapir, et al., 2024) that applied Gestalt principles of closure and figure/ground (Todorovic, 2008) to Hebrew number words. As in Exps. 1 and 2, participants were presented with colored slices and were asked to determine the number of colors presented. Interestingly, in the current experiment, neutral trials were faster than congruent trials. This reverse facilitation, which indicates the existence of task conflict, may reflect the difficulty in perceiving the stimuli and is in line with previous findings that showed evidence for task conflict when the irrelevant dimension was difficult to perceive (Hershman, Sapir, et al., 2024). This evidence for the existence of task conflict in the color-digit Stroop task is also consistent with a previous color-digit Stroop task and pupillometry (Hershman, et al., 2025a) findings that showed evidence for both task and information conflicts in the color-number Stroop task.

## 5. Combined Analysis of Experiments 1, 2, and 3

We next examined whether the differences between congruent and neutral trials were different in the three experiments (The data were analyzed using JASP (JASP Team, 2025)). Mean RTs of correct response trials for each participant in each condition were subjected to a two-way mixed ANOVA with congruency (congruent, incongruent, and neutral) and task (digits, Hebrew number words, and Gestalt-based Hebrew number words) as independent factors. Our analysis suggested no meaningful differences between the experiments regardless of the congruency conditions (*F*(2,109) = 1.26, *p* = .29, \[
\eta _{p}^{2}\] = .023, *BF*_10_ = 0.531 ≡ *BF*_01_ = 1.88). In addition, and as expected, our analysis produced a meaningful (*BF*_10_ ≥ 3) interaction effect, *F*(4,218) = 9.10, *p* < .001, \[
\eta _{p}^{2}\] = .14, *BF*_10_ > 10^5^. Another two-way mixed ANOVA was conducted without the incongruent trials with the aim of verifying that the interaction was caused by the differences between congruent and neutral trials.^1^ As expected, our analysis produced a meaningful (*BF*_10_ ≥ 3) interaction effect, *F*(2,109) = 27.76, *p* <. 001, \[
\eta _{p}^{2}\] = .34, *BF*_10_ > 10^6^. Post-hoc comparisons showed meaningful differences in the facilitation between digits (which were used in Exp. 1) and Hebrew number words (which were used in Exp. 2) (*F*(1,109) = 13.76, *p* < .001, *BF*_10_ = 139.81) and between Hebrew number words (which were used in Exp. 2) and Gestalt-based Hebrew number words (which were used in Exp. 3) (*F*(1,109) = 12.77, *p* < .001, *BF*_10_ = 29.06). Transitively, there were meaningful differences in the facilitation between Gestalt-based Hebrew number words (which were used in Exp. 3) trials and digits (which were used in Exp. 1) trials (*F*(1,109) = 55.38, *p* < .001, *BF*_10_ > 10^7^).

## 6. Experiment 4

To explicitly assess whether the stimuli used in Exps. 1–3 indeed differ in their difficulty levels; another experiment was conducted. In this experiment, participants were presented with number stimuli, which were similar to the ones used in Exps. 1–3, and were asked to respond to their numerical value as quickly as possible. Consistent with the variations observed in the different experiments, we anticipated differences in RTs according to the type of stimuli so that responses to digits will yield the shortest RTs, those to Hebrew number words will result in longer RTs, and RTs to Gestalt-based Hebrew number words will be the longest.

### 6.1. Method

#### 6.1.1. Participants

Similar to Exps. 1–3, we asked for the required sample size for an expected effect size of \[
\eta _{p}^{2}\] = .157 (corresponding to a Cohen’s f = 0.431), for three types of stimuli. With α set at .05 and desired power at .95, the analysis indicated that a total of 17 participants would be required to detect a main effect. However, since the experiment was conducted online, we expected a larger number of submissions and therefore recruited a large number of participants.

Twenty-nine participants (28 females & 1 male, mean age 22.91 years, *SD* = 1.63) from Achva Academic College participated in the experiment in return for course credit. The study was approved by the ethics committee of the Psychology Department of Ben Gurion University. None of the participants reported a history of attention deficit disorder, learning disabilities, or color blindness.

#### 6.1.2. Stimuli

Participants were presented with black (RGB: 0, 0, 0) numbers (1–4) in a size of 357 × 551 pixels. The visual properties of the stimuli were similar to those used in Exps. 1–3, with one exception; all the stimuli were black and white. Specifically, participants were presented with digits, Hebrew number words, and Gestalt-based number words (the entire set of stimuli can be downloaded from: https://osf.io/f27j8/?view_only=c94d9d6d718d4883b30f991c8a2cd1f0). The conditions and the stimuli for each participant were selected randomly from a pool that included all the possible combinations. The presented stimuli appeared against a white (RGB: 255, 255, 255) background.

#### 6.1.3. Procedure

Similar to Exps. 1–3, participants were tested online by using minnoJS (Zlotnick et al., 2015) on their own devices. The program required a spacebar response, ensuring participants only use computers rather than tablets or mobile phones. The experiment included 12 practice trials that were excluded from the analysis. After each practice trial, participants received feedback on their accuracy. Participants had to achieve at least 50% correct trials to proceed to the experimental part (i.e., at least ten correct responses). Failure in practice required a repetition of the practice. In the experimental part, participants were presented with 432 trials; 144 for each type of stimulus. A trial began with 500 ms of a black fixation cross at the center of the screen. The fixation was followed by a visual stimulus that appeared on the screen for 400 ms, and was followed by a blank screen for a maximum of 1,100 ms or until a key-press. Each trial ended with a 1,000-ms ITI of a blank screen. Participants were asked to respond using both hands and press the “Z” key if the numerical value of the stimulus was 1, the “X” key if the numerical value of the stimulus was 2, the “N” key if the numerical value of the stimulus was 3, and the “M” key if it was 4. RT was calculated from the appearance of the visual stimulus to the reaction in the form of a key-press.

### 6.2. Results

Five participants were excluded from the analysis as they had an accuracy lower than 50% in each condition. Similar to Exps. 1–3, for each participant (in total 24 participants; 23 females & 1 male, mean age = 23.1 years, SD = 1.71), mean RT and standard deviation were calculated across all the experimental trials. Then, RTs that were larger or smaller than 2.5 z-scores from the mean of each subject were excluded from the analysis. Overall, 12.95% of trials were excluded. The data were analyzed using JASP (JASP Team, 2025).

#### 6.2.1. Error Rates

The mean error rates for each participant in each condition were subjected to a one-way repeated-measures ANOVA with stimulus type (digits, Hebrew number words, and Gestalt-based Hebrew number words) as an independent factor (mean error rates in the various conditions are presented in Table 2). The analysis produced a meaningful (*BF*_10_ ≥ 3) main effect for stimulus type, *F*(2,46) = 20.70, *p* < .001, \[
\eta _{p}^{2}\] = .47, *BF*_10_ > 10^4^. Specifically, mean error rates in Gestalt-based Hebrew number words trials were higher than in Hebrew number words trials, *t*(23) = 3.83, *p_holm_* = .002, *BF*_10_ = 49.55, *Cohen’s d* =0.78, which were higher than in digits trials, *t*(23) = 2.84, *p_holm_* = .009, *BF*_10_ = 5.18, *Cohen’s d* = 0.58. Transitively, Gestalt-based Hebrew number words trials showed more error rates than digits trials *t*(23) = 5.56, *p_holm_* < .001, *BF*_10_ > 1,000, *Cohen’ s d* = 1.13.

#### 6.2.2. Reaction time

Mean RTs of correct response trials for each participant in each condition were subjected to a one-way repeated-measures ANOVA with stimulus type (digits, Hebrew number words, and Gestalt-based Hebrew number words) as an independent factor (mean RTs in the various conditions are presented in Table 2). As expected, our analysis produced a meaningful (*BF*_10_ ≥ 3) main effect for stimulus type, *F*(2,46) = 135.56, *p* < .001, \[
\eta _{p}^{2}\] = .85, *BF*_10_ > 10^16^. Specifically, mean RT in Gestalt-based Hebrew number words trials was longer than in Hebrew number words trials, *t*(23) = 8.40, *p_holm_* < .001, *BF*_10_ > 10^5^, *Cohen’s d* = 1.71, which were longer than in digits trials, *t*(23) = 9.25, *p_holm_* < .001, *BF*_10_ > 10^6^, *Cohen’s d* = 1.89. Transitively, mean RTs in Gestalt-based Hebrew number words trials were longer than in digits trials *t*(23) = 14.61, *p_holm_* < .001, *BF*_10_ > 10^10^, *Cohen’s d* = 2.98.

### 6.3. Discussion

In this experiment, we presented participants with the same stimuli used in Exps 1–3 and instructed them to respond based on the numerical values of these stimuli. We aimed to assess how difficult it was to process each stimulus. As expected, the results indicated more effort for Gestalt-based Hebrew number words than for Hebrew number words, which in turn required more effort than digits. These results, which are in line with previous studies that showed that digits are easier to process than words (Brysbaert, 2005; Schubert, 2017), support our hypothesis regarding the varying levels of difficulty of processing the three types of stimuli.

## 7. General Discussion

In the present study, we used the color-digit Stroop task (Hershman, et al., 2025a, 2025b) while varying the difficulty of processing the irrelevant dimension. Participants were required to determine, as fast as possible, how many colors were presented on the screen and ignore the meaning of the stimuli (if there was any). The presented stimuli could be meaningless (e.g., slices of colors), or they could include numerical values (e.g., single digits). The numerical meaning of the stimuli could be congruent with the number of the presented colors (e.g., the digit ‘3’ on slices with three colors) or incongruent with the number of presented colors (e.g., the digit ‘2’ on slices with three colors).

Across three experiments, we varied the difficulty of processing the irrelevant dimension of the stimuli (i.e., their irrelevant numerical value). In Exp. 1, when the irrelevant dimension — single white digits presented at the center of the colored slices — was easy to perceive, both interference and facilitation were observed. In Exp. 2, Hebrew number words cut into four slices in different colors were used, and only interference was found (without facilitation). In Exp. 3, when the Hebrew number words were presented implicitly using Gestalt principles, congruent trials were faster than incongruent trials but slower than neutral trials. This reverse facilitation indicates the existence of task conflict (i.e., processing of the numerical value of the stimulus vs. counting the number of colors) in addition to the frequently found information conflict.

The results of Exp. 1 replicated those of previous color-digit Stroop studies (Hershman, Keha, et al., 2024; Hershman, et al., 2025a, 2025b). These findings, comprising both interference and facilitation effects, demonstrate the automaticity of numerical processing. Specifically, they support the hypothesis that numerical processing holds evolutionary significance (Feigenson et al., 2004), as evidenced by the task-irrelevant processing of the numerical meaning of the stimulus during task-relevant counting.

The results of Exp. 3 extend our knowledge about task-irrelevant processing of the numerical meaning. In line with a previous color-digit Stroop task and pupillometry study (Hershman, et al., 2025a), our results support the hypothesis regarding the existence of task conflict in the color-digit Stroop task (i.e., processing of the numerical value of the stimulus vs. counting the number of colors that consisted of the stimulus), in addition to the existence of information conflict (i.e., the numerical value given by the meaning of the stimulus vs. the number of colors of the stimulus). Similar to the color-word Stroop task, these findings suggest that stimuli activate tasks that are associated with them (Kiesel et al., 2007; Meiran & Kessler, 2008; Rogers & Monsell, 1995; Waszak et al., 2003; Wendt et al., 2013). Words (as well as digits) tend to automatically evoke reading (Monsell et al., 2001), and as a result, when counting the number of presented colors is required, the automatically evoked reading task competes with the counting task and creates a task conflict. This means that conditions that consist of two tasks (both congruent and incongruent) result in more mental effort than conditions that only consist of one task (neutral trials), which is indicated by reverse facilitation (Kalanthroff et al., 2018; Keha & Kalanthroff, 2023). While reverse facilitation is often found by using measurement of brain activation (Aarts et al., 2009; Bench et al., 1993; Carter et al., 1995) and changes in pupil size (Hershman et al., 2020, 2021; Hershman & Henik, 2019, 2020), it is rarely seen in RT experiments and can be observed only under certain conditions, for example, when the expectation for conflict is low (Goldfarb & Henik, 2007), in a stop-signal task (Kalanthroff et al., 2013), and when preparation time is reduced in a task-switching task (Kalanthroff & Henik, 2014). In our study, in line with Hershman et al.’s findings (Hershman, Sapir, et al., 2024), reverse facilitation, as evidence for task conflict, was found only when the processing of the irrelevant dimension (i.e., the numerical value given by the meaning of the stimulus) was difficult to process (Exp. 3).

In Exp. 2, processing of the irrelevant dimension (i.e., the numerical value given by the meaning of the stimulus) was intermediate in difficulty: it was less challenging than the irrelevant stimuli in Exp. 3, but more so than the irrelevant stimuli in Exp. 1. Interestingly, in this case, no meaningful facilitation was found. This shift from facilitation to reverse facilitation supports the hypothesis that the difficulty level of processing the irrelevant dimension contributes to task conflict. That is, the more difficult it is to perceive the irrelevant dimension, the more prominent the task conflict becomes. It is important to note that there is evidence in the literature showing reverse facilitation in the color–word Stroop task without lowering task control or manipulating difficulty (Entel et al., 2015; Schulz, 1979; Spinelli & Lupker, 2025). Although these findings seem to challenge the interpretation of facilitation as a marker of task conflict, the current results showing a clear modulation of facilitation as a function of task-irrelevant difficulty are consistent with the view that facilitation reflects variations in task conflict, as supported by many previous studies (see Kalanthroff et al., 2018 for a review).

Based on these changes, progressing from facilitation to reverse facilitation, as a function of the difficulty of the irrelevant task, we suggest that task conflict exists even when there is no evidence for it. Specifically, we posit that at any given time, during a Stroop (or Stroop-like) task, there are two competing processes: the facilitation from the congruent stimuli and the cost of performing two tasks. The observed facilitation suggests that the reading can facilitate our performance in the case of two supportive pieces of information (Augustinova et al., 2019; MacLeod, 1991). However, reverse facilitation (Goldfarb & Henik, 2007; Hershman, Sapir, et al., 2024; Hershman & Henik, 2019) suggests that the completion of the irrelevant task may require additional effort compared to performing a single task.

Another relevant discussion in the literature concerns whether Stroop facilitation is driven by inadvertent reading (MacLeod & MacDonald, 2000) or by converging information from the color and the word dimensions (Roelofs, 2010). According to the inadvertent reading theory, since reading is automatic, in a congruent condition, the automatic process of reading leads to facilitation (MacLeod & MacDonald, 2000). In contrast, the converging information theory ruled out this idea and suggested that the convergence of the information from the color and the word is the primary source that drives this effect (Roelofs, 2010). Thus, one might suggest that the convergence of information that facilitates response might hide the fact that inadvertent reading took place and also slowed down response as a result of the automatic reading process. In this sense, the apparent facilitation may be somewhat misleading—it may not reflect a pure benefit but rather a composite of opposing influences: a genuine convergence-based speed-up and an underlying cost of automatic word processing. That is, even in cases where congruent stimuli produce faster responses, this does not necessarily mean that reading was successfully suppressed; instead, it may indicate that reading occurred automatically, introduced some degree of conflict, and yet the overlap between the word and color dimensions compensated for that cost.

Our results suggest that what determines which process will dominate is the relative difficulty of the irrelevant task. When the irrelevant task (e.g., reading) requires substantially more time or cognitive resources due to its difficulty, task conflict is more likely to emerge. It is also important to note that part of the observed facilitation might actually reflect instances of failure to fully engage with the relevant task—failures that would go unnoticed when the stimulus is congruent. In line with the notion of task conflict, once the irrelevant task of reading is automatically initiated and completed, the resulting processing may either interfere with response selection in incongruent trials or appear to facilitate it in congruent ones. However, as suggested by the discussion above, such apparent facilitation might be somewhat misleading: it may not represent a pure advantage but rather the net outcome of two opposing forces—a genuine convergence-based speed-up and a cost associated with the automatic activation of the reading process. In this view, facilitation effects do not necessarily indicate successful suppression of reading but may instead reveal the unavoidable influence of automatic word processing, whose impact is masked when word and color dimensions converge on the same response. That is, facilitation reflects a failure in the completion of the relevant task. This failure to complete solely the relevant task is the basic concept behind task conflict (Hershman & Henik, 2019; Littman et al., 2019).

An alternative explanation for the differences observed between the experiments could be that they were driven by the relevant task itself, specifically, by variations in its difficulty level across experiments. As the task became more difficult from Exp. 1 to Exp. 3, responses to both congruent and incongruent stimuli may have slowed accordingly. In contrast, responses to neutral stimuli would remain relatively stable or slow down much less, since the neutral stimuli were identical across all three experiments. However, although RTs tended to be longer from Exp. 1 to Exp. 3, accompanied by a trend toward more errors, a direct comparison of RTs between the experiments (across all conditions) showed no meaningful differences. Hence, while changes in the difficulty of the relevant dimension might account for overall longer RTs across experiments, as might be suggested by the general RT trend, they cannot explain why the differences were specifically associated with task conflict (i.e., the difference between neutral and congruent trials) while the interference effect remained unchanged (i.e., the difference between neutral and incongruent trials). In addition, and importantly, the difficulty levels of the relevant dimension cannot explain the results in the Gestalt color-word Stroop task (Hershman, Sapir, et al., 2024), where participants had to name the color of a single-color stimulus, just like in any other Stroop task. In that study, participants were presented with a Gestalt stimulus, similar to the one presented in Exp. 3, but which only contained one color, and were asked to name the color of the stimulus. The difficulty of the relevant task was equivalent to that of other Stroop tasks, but, similar to the present study, reverse facilitation was observed.

It might be suggested that different types of neutral (such as letter strings, symbols, or shapes) might be used with the aim of rejecting any perceptual differences between the neutral and the congruent condition that might explain the differences between the conditions. However, similar to the Gestalt color-word Stroop task (Hershman, Sapir, et al., 2024), we aimed to use a neutral that would not evoke task conflict (Hershman et al., 2020, 2021). Hence, we chose colored rectangles as neutrals. It is possible that further studies that will examine different types of neutral stimuli in the color-digit Stroop task, as well as in the Gestalt color word Stroop task (Hershman, Sapir, et al., 2024), might investigate the interaction between the difficulty level of the irrelevant dimension and the meaningfulness level of it.

Another possible limitation that could be addressed in future studies is that in the current study, the difficulty levels of the irrelevant task were not examined within the same experiment. Therefore, it could be argued that other parameters could affect the differences in the manifestation of task conflict between the three experiments. However, it is important to note that even though we did not include a direct manipulation of task difficulty, the task itself was identical in all three experiments in terms of the required task (color naming) and the procedure of the study, and only differed in the stimuli that were used (except for the identical neutral stimuli that were used across the three experiments). Nevertheless, future studies could elaborate on the results of the present study by including a manipulation that will test the effect of difficulty on task conflict within the same experiment using the exact same stimuli with slight variations in difficulty levels of the irrelevant dimension.

The findings from the present study are consistent with the disfluency effect, which posits that increasing the difficulty of processing a stimulus can enhance cognitive engagement (Alter et al., 2007; Seufert et al., 2017). In the context of the Gestalt-color-digit Stroop task, our results indicate that when the irrelevant dimension becomes harder to perceive, task conflict intensifies, as demonstrated by reverse facilitation (Hershman, Sapir, et al., 2024). This pattern aligns with the notion that perceptual challenges can prompt deeper cognitive processing rather than being automatically dismissed. Therefore, the increased difficulty of the irrelevant dimension in our experiments likely triggered a shift from automatic to more controlled processing, which amplified task conflict. This insight extends the disfluency effect by demonstrating that difficulty in processing irrelevant information can increase, rather than decrease, cognitive engagement, even when that information is not essential, and can even disrupt task performance. Increasing the difficulty of the irrelevant dimension even more (e.g., by using a contrast that makes the text even harder to read) could lead to extreme disfluency. This may impair performance by overloading extraneous cognitive load (Diemand-Yauman et al., 2011; Seufert et al., 2017). In such cases, participants are unlikely to process the irrelevant dimension, and performance might follow a reverse U-shaped pattern (Oppenheimer & Alter, 2014).

This pattern aligns with the view that task conflict reflects a dynamic and differentiated competition between task sets, shaped by factors such as habit strength and cognitive resource allocation. Reading, in particular, is not only reflexively triggered but also remarkably persistent: even degraded or peripheral inputs continue to elicit processing attempts. In the present study, the habit strength of reading causes more cognitive resource allocation once the irrelevant task is available. Moreover, this habit strength requires more cognitive resource allocation as a function of the difficulty level of the irrelevant dimension.

This interpretation is supported by findings from Cohen et al. (2008), who demonstrated that when word stimuli are degraded (through rotation, spacing, or peripheral presentation), the ventral reading pathway becomes insufficient. In such cases, the brain recruits dorsal attention networks to support lexical processing through more effortful, top-down mechanisms. Thus, increased difficulty does not necessarily lead to disengagement; instead, it often prompts the cognitive system to invest additional resources in attempting to process irrelevant stimuli, even when doing so is counterproductive.

Why would a difficult stimulus, especially if it is not task-relevant, be processed at all? It could be argued that words with reduced readability will be effortlessly dismissed and, thus, result in a smaller information conflict and a small or non-existent task conflict. However, in contrast with this intuitive suggestion, it has been shown that participants who completed the color word Stroop task in their non-native language did not show these intuitive results (Datta et al., 2019; Tzelgov et al., 1990) and rather showed evidence for a larger information conflict. Furthermore, this also does not seem to be the case for participants with reading difficulties either (Protopapas et al., 2007), which again revealed a larger information conflict instead of a reduced one. This implies that participants with reduced ability to read still complete the automatic task of reading, even when it requires additional cognitive resources.

The existence of information conflict in Exps. 1–3 suggests that our ability to inhibit the involuntary activation of an unwanted process is restricted (Monsell et al., 2001; Stroop, 1935). Therefore, our results demonstrate that despite the resource-intensive nature of completing the irrelevant task of reading, it still takes place. In that sense, reading is an involuntary process that is automatic (Augustinova & Ferrand, 2014; MacLeod, 1991; Stroop, 1935). However, the presence of task conflict as a function of the difficulty of the irrelevant task implies that reading requires cognitive resources and, as a result, cannot be referred to as a completely automatic process in terms of the usage of cognitive resources (Schneider & Chein, 2003). Therefore, our finding suggests that while reading is considered an involuntary and automatic process, its execution is not entirely devoid of cognitive resource allocation.

## Summary

The present study was conducted with the aim of exploring key issues surrounding task conflict and cognitive control. In Exp. 1, we successfully replicated prior findings using the color-digit Stroop task (Hershman, et al., 2025b), thereby strengthening confidence in both the robustness and reliability of this paradigm. This replication not only confirms earlier results but also reinforces the color-digit Stroop task as a powerful and dependable tool for advancing future research in this domain. Second, we expanded the scope of task conflict by showing that the semantic content of an irrelevant dimension can impact performance (Exp. 3), even within a paradigm markedly different from the classic color-word Stroop task (Hershman, Sapir, et al., 2024). This result underscores the broader applicability of task conflict effects, revealing their relevance across diverse forms of cognitive processing. Third, and perhaps most notably, this study investigated the dynamic nature of task conflict. Across three core experiments, along with an explicit examination in Exp. 4, we systematically varied the difficulty of the irrelevant dimension. The findings revealed that task conflict is not an all-or-none phenomenon. Instead, it increases progressively as the complexity of the irrelevant information rises, as reflected in the contrast between congruent and neutral trials.

Taken together, these three contributions offer valuable insights into the mechanisms underlying cognitive control. Our findings suggest that as the difficulty of the irrelevant task increases, task conflict to complete the relevant task also increases. We assume, however, that this effect would diminish once the irrelevant task becomes much more difficult than the difficulty level examined in the current study. These results not only affirm the reliability and validity of our methodological approach but also expand the theoretical framework of task conflict. In doing so, they open new avenues for research across related areas in cognitive psychology and cognitive neuroscience, where understanding the interplay between competing task demands remains a central challenge.

## Data Accessibility Statement

Data for the experiment is publicly available on OSF at: https://osf.io/f27j8/?view_only=c94d9d6d718d4883b30f991c8a2cd1f0.

## Footnotes

Another two-way mixed ANOVA was conducted without the congruent trials, confirming that the interference effects were statistically identical across the three experiments (as it is reflected in *BF*_01_ > 3).
